# Dendritic tree extraction from noisy maximum intensity projection images in *C. elegans*

**DOI:** 10.1186/1475-925X-13-74

**Published:** 2014-06-12

**Authors:** Ayala Greenblum, Raphael Sznitman, Pascal Fua, Paulo E Arratia, Meital Oren, Benjamin Podbilewicz, Josué Sznitman

**Affiliations:** 1 Department of Biomedical Engineering, Technion - Israel Institute of Technology, 32000, Haifa, Israel; 2 School of Computer and Communications, Ecole Polytechnique Fédérale de Lausanne (EPFL), 1015, Lausanne, Switzerland; 3 Department of Mechanical Engineering and Applied Mechanics, University of Pennsylvania, 19104, Philadelphia, USA; 4 Department of Biology, Technion - Israel Institute of Technology, 32000, Haifa, Israel; 5 Current address: Department of Biochemistry & Molecular Biophysics, Columbia University, 1032, New York, USA

**Keywords:** Neuronal dendrites, *C. elegans*, Computer vision, Image segmentation, Statistical learning, Bayesian probability, Neuronal arborization

## Abstract

**Background:**

Maximum Intensity Projections (MIP) of neuronal dendritic trees obtained from confocal microscopy are frequently used to study the relationship between tree morphology and mechanosensory function in the model organism *C. elegans*. Extracting dendritic trees from noisy images remains however a strenuous process that has traditionally relied on manual approaches. Here, we focus on automated and reliable 2D segmentations of dendritic trees following a statistical learning framework.

**Methods:**

Our dendritic tree extraction (DTE) method uses small amounts of labelled training data on MIPs to learn noise models of texture-based features from the responses of tree structures and image background. Our strategy lies in evaluating statistical models of noise that account for both the variability generated from the imaging process and from the aggregation of information in the MIP images. These noisy models are then used within a probabilistic, or Bayesian framework to provide a coarse 2D dendritic tree segmentation. Finally, some post-processing is applied to refine the segmentations and provide skeletonized trees using a morphological thinning process.

**Results:**

Following a Leave-One-Out Cross Validation (LOOCV) method for an MIP databse with available “ground truth” images, we demonstrate that our approach provides significant improvements in tree-structure segmentations over traditional intensity-based methods. Improvements for MIPs under various imaging conditions are both qualitative and quantitative, as measured from Receiver Operator Characteristic (ROC) curves and the yield and error rates in the final segmentations. In a final step, we demonstrate our DTE approach on previously unseen MIP samples including the extraction of skeletonized structures, and compare our method to a state-of-the art dendritic tree tracing software.

**Conclusions:**

Overall, our DTE method allows for robust dendritic tree segmentations in noisy MIPs, outperforming traditional intensity-based methods. Such approach provides a useable segmentation framework, ultimately delivering a speed-up for dendritic tree identification on the user end and a reliable first step towards further morphological characterizations of tree arborization.

## Background

**Figure 1:**
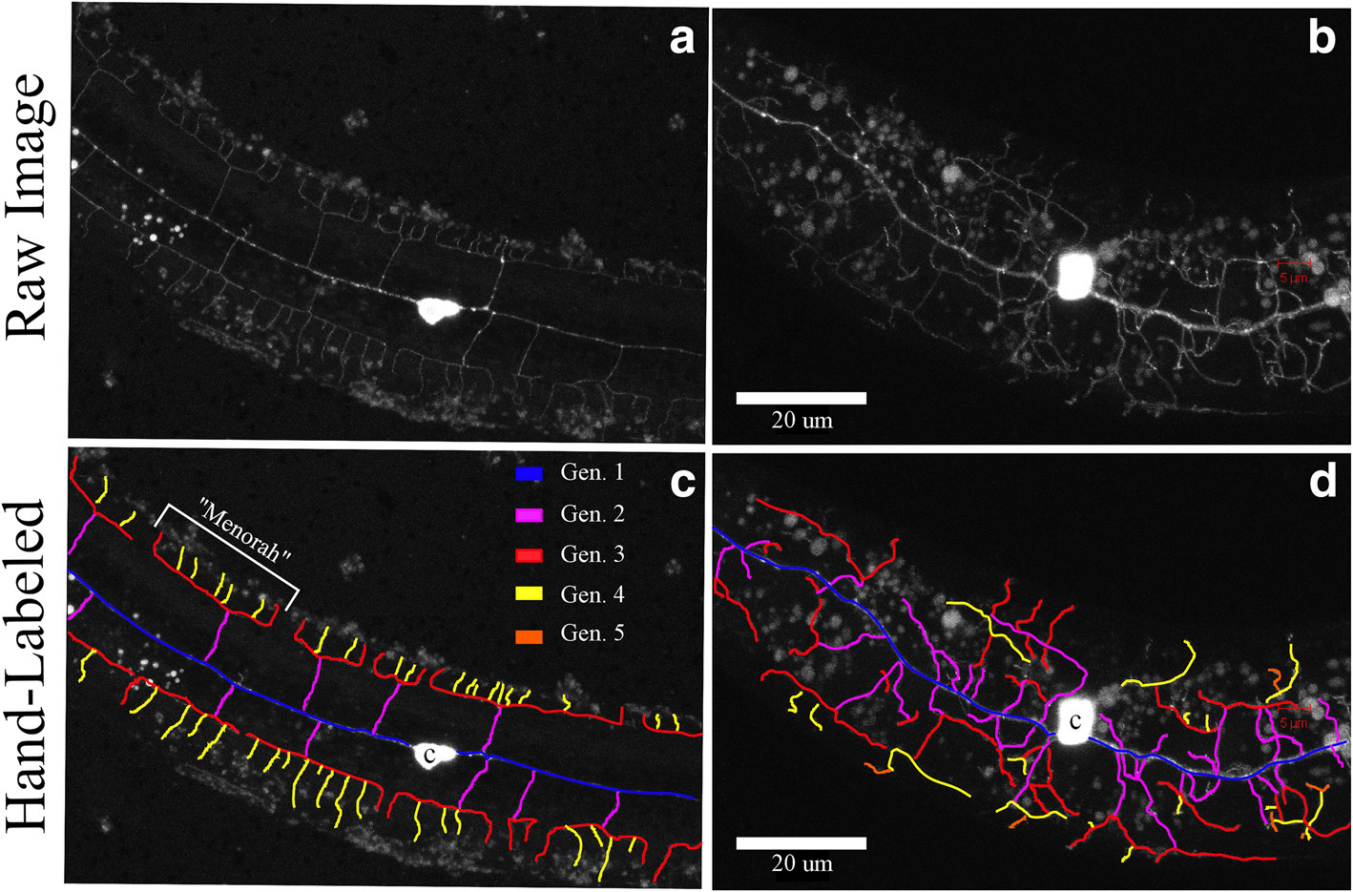
**Mechanosensory PVD neuronal dendrites in the nematode *C. elegans*.** Examples of **(a,b)** original MIP and **(c,d)** manually-delineated mechanosensory PVD neuronal dendrites in the nematode *C. elegans*, including annotated branching generations. MIP of **(a)** wild-type dendritic PVD tree and **(b)** disorganized PVD arborization with mutations in cell fusion gene *eff*-1, where nematodes show reduced sensitivity to strong mechanical stimuli [6]. Wild-type dendritic PVD trees show repetitive structural units known as “menorah” [6]. “c” denotes cell body (soma) and small droplets are autofluorescent gut granules. Note that additionally an axon goes out from the cell soma which is not visualized here.

Organisms perceive their surroundings primarily through sensory modalities including the perception of touch. Touch is anatomically complex and involves the largest organ of the body [1], namely the skin. Organisms critically depend on touch for daily activities including feeding, locomotion and communication amongst other. To convey touch-based sensory inputs, neurons must have the ability to sense and translate mechanical stimuli such as force, pressure, stress, into electrical signals; this process is known as mechanosensation. Many mechanosensory functions rely on the proper structuring and development of neuronal dendritic trees (or arborization), as shown in Figure 1a, where morphological patterns of dendritic trees ultimately determine how a neuron processes its mechanosensory input [2,3]. In particular, defective dendritic arborization (see example in Figure 1b) is acknowledged to contribute to neuro-developmental disorders [1,4]. How mechanosensory functions are linked to dendritic tree morphologies, and vice-versa, is of pivotal concern in developmental biology [5,6]. Hence, an integral characterization of the relationship and dependency between mechanosensory input and dendritic arborization is essential towards a better understanding of neuro-degenerative diseases and potential treatment strategies.

To study the relationship between tree morphology and mechanosensory function, images of mechanosensory neurons are typically acquired using laser scanning confocal microscopy (Figure 1), where neurons are fluorescently labelled (e.g., GFP). For example, this is done for the ubiquitous nematode *Caenorhabditis elegans*[6]; this small (approximately 1 mm long), free-living nematode is widely used as a model organism for biological research [7-9]. In particular, *C elegans* relies on chemosensation to interact with its surrounding environment and, importantly on mechanosensory touch neurons (e.g., PVD and FLR) for avoidance responses (e.g., stay away from potential threats [10]) and proprioception (i.e., sensing the relative position of neighbouring body parts including body angle [5]). The advantages of using *C. elegans* critically include the knowledge of the complete developmental program of cell division and connectivity pattern of its neurons [11,12]. Moreover, *C. elegans* is an attractive candidate to study mechanosensation since its nervous system includes sensory neurons that are both morphologically and functionally similar to those in humans [13], with dendrites positioned underneath the nematode cuticle (i.e., skin) between its epidermis.

From an imaging point of view, the choice of confocal microscopy, including spinning disk confocal (SDC) microscopy, is advantageous as it allows for high-resolution imaging of complex internal structures in *C. elegans*. Such imaging modalities deliver images to the user via three-dimensional (3D) volumes or Maximum Intensity Projections (MIP) images. The latter constitute projections, or accumulation of responses, of 3D-stacks visualized onto a two-dimensional (2D) image (see examples of Figure 1a,b). Compared to their 3D counterparts, MIPs have the benefit of requiring far less memory and allow large test arrays to be performed without the need for excessive storage systems. In particular, since the landmark study of Meital *et al.*[6], there has been a recent proliferation in works on the characterization of dendritic tree arborization in *C. elegans* using MIPs [5,14-20].

Generally, the process of building morphological arborization models of neurons includes amongst other the quantification of the total number of dendrites (e.g., primary, secondary, tertiary generation), as shown in Figure 1c,d. This task almost unanimously requires first extracting the shape of dendritic trees from binary images (or *segmentations*) to separate the tree structure from its environment (i.e., *background*), and referred here as dendritic tree extraction (DTE). Traditionally, the characterization of dendritic trees has relied on cumbersome manual approaches for delineation, resulting in a slow and strenuous process that can take up to months for entire datasets [21]. Hence, given the abundance of potential users there is an ongoing need to develop automated algorithms that can first extract morphological structures of neuronal dendrites.

A wide array of methods have been proposed to automatically segment 3D trees from image stacks. In most cases, these methods start by applying some level of image processing such as image intensity thresholding, edge or texture extraction, or path classification to provide some coarse or over-complete representation of the tree structure in the image stacks. In particular, intensity-based approaches segment images by defining a threshold value (or several values) that will separate tree structures from their background [22,23]. These methods are particularly suitable for images where tree pixels present homogenous brightness that are consistently different from homogenous background pixels. Alternatively, Texture-Based approaches segment images according to regions with different texture features [24,25], with texture defined as the spatial arrangement of a group of pixels representing a sub-pattern, arranged in a more or less periodic manner [26].

From such representations, a tree model is then imposed to add or remove elements from the initial representation and provide a coherent final tree segmentation. Popular methods often belong to either *tracing*[27] or *skeletonization*-based methods [28] that make use of local structure for segmenting. The former method uses the 3D tube-like local geometry of a tree while the latter relies on the geometric medial axis of the data to capture the neuronal topology. In the context of tree tracing, semi-automatic methods such as in the work of Peng *et al.*[29] have been shown to be highly appropriate. For such approaches, a user first selects a set of seed vertices which the final tree tracing should include, and then a local or global optimization schemes connects vertices by using the image data. These methods have been shown to be highly effective in challenging 3D image stacks, using little to moderate manual user input. Other strategies which are fully automatic and require virtually no user input, outside of a training phase (*i.e.* requiring labeled training data), have also been proposed [30-32]. Here, rather, tree traces are achieved by constructing a set of plausible trees and selecting the best one according to maximized global features which leverage training data. Lastly, the recent work of Xiao and Peng [33] provides an automatic tracing method, using no training data at all and few seed points, in order to extract dendritic structures from 3D image data.

While the aforementioned methods have shown to be effective in reconstructions of dendritic trees from 3D image stacks [27,28,34], their application on MIP images is far less convincing. This is in part due to two characteristics of MIP images. To begin, 3D information is overwhelmingly lacking in MIPs to disambiguate overlapping or disappearing curvi-linear structures. This latter point is crucial in a number of successful 3D methods. Additionally, MIPs are typically far more noisy since they represent the accumulation of responses across image stacks. As a result, achieving DTE in *C. elegans* is often more challenging, notably in the presence of intrinsic artifacts including autofluorescent gut granules (see exampes in Figure 1a,b) or nutrient bacteria *E. coli*.

With this in mind, the present article focuses on the automated segmentation of neuronal dendritic trees to provide a reliable alternative to traditional manual approaches in MIP images. Our approach uses small amounts of labelled training data on MIPs to learn noise models of texture feature responses of tree structures and background. In effect, the noise models account for both the variability generated from the imaging process and from the aggregation of information in the MIP images. These noisy models are then used within a probabilistic, or Bayesian framework to provide a coarse 2D dendritic tree segmentation. Finally, some post-processing is applied to refine the segmentation. We show that this approach allows reliable segmentation in a number of imaging conditions, outperforming traditional intensity-based methods for equivalent MIP images. We also demonstrate how our approach compares qualitatively to a state-of-the-art semi-automatic tracing method [29] and furthermore illustrate cases where our approach fails to provide satisfactory segmentations and skeletons as a result of varying imaging conditions.

In the following sections, we first outline our DTE method and probabilistic framework in detail. We then evaluate the performance of our approach on MIPs acquired from confocal imaging using a cross-validation method and compare our segmentation results to existing traditional segmentation methods. Finally, we test our DTE method on examples of independent, previously unseen MIPs and discuss some ongoing limitations.

## Methods

We detail below the proposed DTE framework. Our method relies principally on texture-based image features that are invariant to orientation changes in order to capture tree delineations, thus providing feature that are noisy but yet informative. The resulting noisy features are modelled statistically and serve as the basis for extracting tree structures within a Bayesian framework.

### Notation and model

To begin, we consider each pixel *q* of an image to have a corresponding binary label *L*_*q*_ ∈ {0, 1} indicating that the pixel belongs to the dendritic tree (*L*_*q*_ = 1) or instead to the background (*L*_*q*_ = 0). Given the uncertainty surrounding the value of a pixel label in an image where a tree structure must be extracted, we consider *L*_*q*_ to be a binary random variable with probability distribution *P*(*L*_*q*_).

To determine the value of the pixel label, we can compute image features $X_{q}\in R^{d}$ from pixel *q* and its surroundings. These features characterize the value of the pixel label but are subject to noise in an MIP image. Hence, we consider *X*_*q*_ to be a random variable such that

(1) $$P(X_{q}=x|L_{p})=\left\{\begin{array}{ll} N(x;\mu_{1},\Sigma_{1}), & \text{if}\;L_{p}=1\\ N(x;\mu_{0},\Sigma_{0}), & \text{if}\;L_{p}=0 \end{array}\right)$$

where N(·) is Gaussian probability distribution and the corresponding parameters $\mu \in R^{d}$ and $\Sigma \in R^{d\times d}$ are the corresponding mean and covariance, respectively. How these parameters are estimated is explicitly described in the subsection below (see Model parameter estimation).

To infer the label value of a pixel *L*_*q*_ from the feature vector *X*_*q*_ computed at pixel *q*, we compute the posterior probability

(2) $$\begin{array}{ll} P(L_{p}|X_{q}=x) & =\frac{P(X_{q}=x|L_{p})P(L_{p})}{P(X_{q}=x)},\\ & =\frac{P(X_{q}=x|L_{p})P(L_{p})}{\underset{l=0,1}{\sum }P(X_{q}=x|L_{p}=l)P(L_{p}=l)}. \end{array}$$

As such, our model states that the likelihood of observing a label is proportional to the product of some prior belief (i.e., *P*(*X*_*q*_ = *x*)) and the likelihood of observing a feature vector when the label is known, as introduced in Eq. (1).

### Image features

In MIP images as depicted in the examples of Figure 1, dendritic tree structures can be characterized as curvilinear structures. At a local scale, these structures appear to be tubular-like, positioned in a variety of orientations, and exhibiting different contrast and noise levels. As such, defining good dendritic tree features in MIPs should be invariant to orientation and capable of capturing the tubular nature of the tree branches.

**Figure 2:**
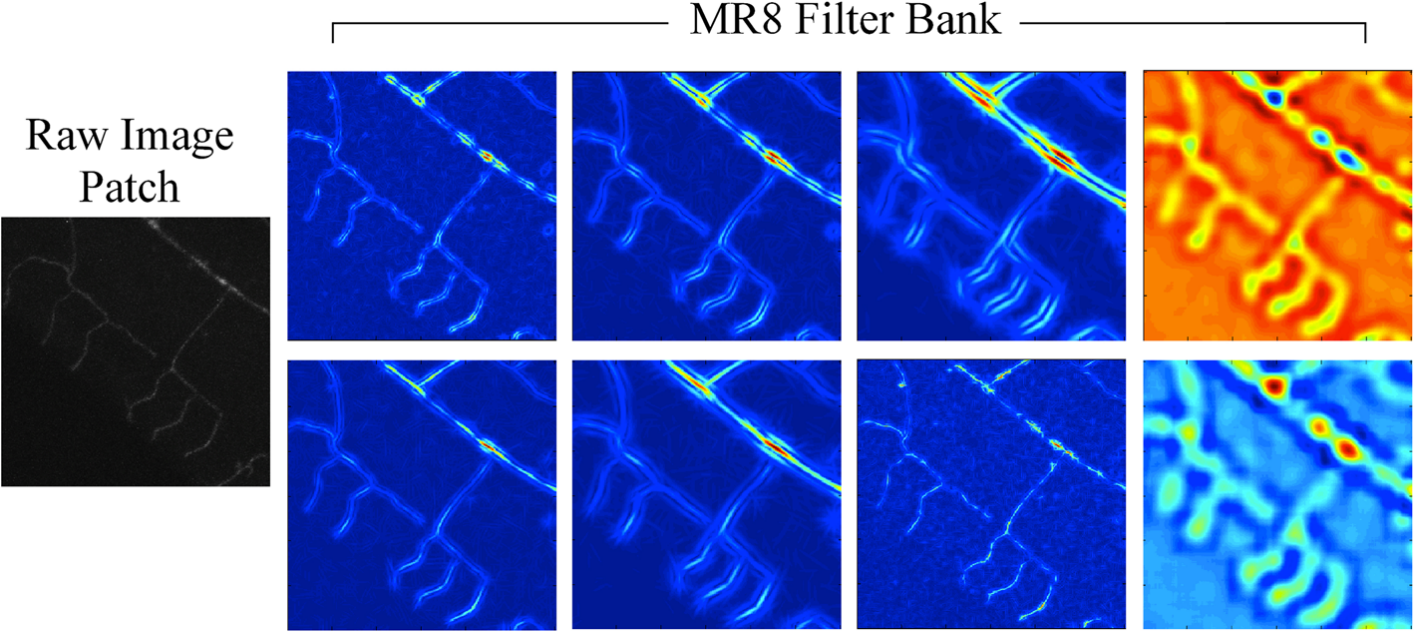
**Illustration of image features.** For a sample image patch of a dendritic tree structure (left), we show the corresponding response images of the 8 filters in the MR8 filter bank. The first three columns depict the response of the two anisotropic filters at three scales. The last column reflects the response of the two isotropic filters. At each pixel, the associated feature vector computed consists of the scalar value at the corresponding location in each of the 8 feature maps.

Given these characteristics, an attractive image feature that appears well suited for such tasks is the Maximum Response 8 (MR8) filter bank [35]. MR8 is directly derived from the Root Filter Set (RFS) [36,38] that computes 38 texture filters using a mixture of anisotropic edge and bar filters, as well as two rotationally symmetric filters, i.e., Gaussian and Laplacian of Gaussian (LoG) filters with different parameters. Hence, the MR8 filter bank maximizes the anisotropic filters’ responses over the six orientations of the RFS absolute responses. This effectively delivers 8 filters (3 scales for the 2 anisotropic filters, in addition to 2 isotropic filters) that are rotational invariant [35] and captures tubular structures of different scale or width. Each of the 8 filters can be applied to an MIP by means of 2D convolutions, resulting in an 8-dimensional feature vector at each pixel location. That is, we let $X_{q}\in R^{8}$ correspond to the feature extracted at location *q*, as depicted in the sample image patch of Figure 2. Note that here we used the implementation provided by Leung and Malik [36] for our image bank filter [37] and thus used their filter parameters for all conducted experiments.

### Model parameter estimation

To estimate the parameters of the noise models, $N(\cdot ;\mu_{1},\Sigma_{1})$ and $N(\cdot ;\mu_{0},\Sigma_{0})$, we make use of a training set, $T=\{(I_{0},S_{0}),\ldots ,(I_{N},S_{N})\}$, where *I*_*i*_ is an image with a dendritic tree in it and *S*_*i*_ is a binary image (or “mask”) with pixels equal to 1 at all locations where the dendritic tree appears. *S*_*i*_ are generated manually by a human expert, i.e., the human-user annotates each pixel where a dendritic tree appears in an image; this set of annotations is often called “ground truth”.

In order to determine parameters of our features models, we randomly sample 30% of pixels that belong to the background and the same percentage of pixels that belong to the foreground. These pixels are selected using the manually-segmented “ground truth” binary images, ${\left\{S_{i}\right\}}_{i=0}^{N}$. The samples’ locations are chosen uniformly at random for each image. Next, we compute the image feature at each sampled image location. To estimate (*μ*_1_,*Σ*_1_), we make use of only the image features that were selected from dendritic tree locations and compute the Maximum Likelihood Estimator (MLE) [39] for both *μ*_1_ and *Σ*_1_. Similarly, we use only background samples to estimate (*μ*_0_,*Σ*_0_), as was done for (*μ*_1_,*Σ*_1_).

### Tree extraction

At testing time, we are interested in extracting the dendritic tree for a new given image. First, we compute the MR8 image features at each pixel location of the image. Next, we evaluate Eq. (2) for each pixel in the MIP using the image features and the models learned from the training data. In particular, we assume the prior *P*(*L*_*q*_ = 1) = *P*(*L*_*q*_ = 0) = 1 / 2, thus allowing us to simplify Eq. (2) to

(3) $$\begin{array}{ll} P(L_{p}=1|X_{q}=x) & =\frac{P(X_{q}=x|L_{p}=1)}{P(X_{q}=x|L_{p}=0)+P(X_{q}=x|L_{p}=1)}. \end{array}$$

**Figure 3:**
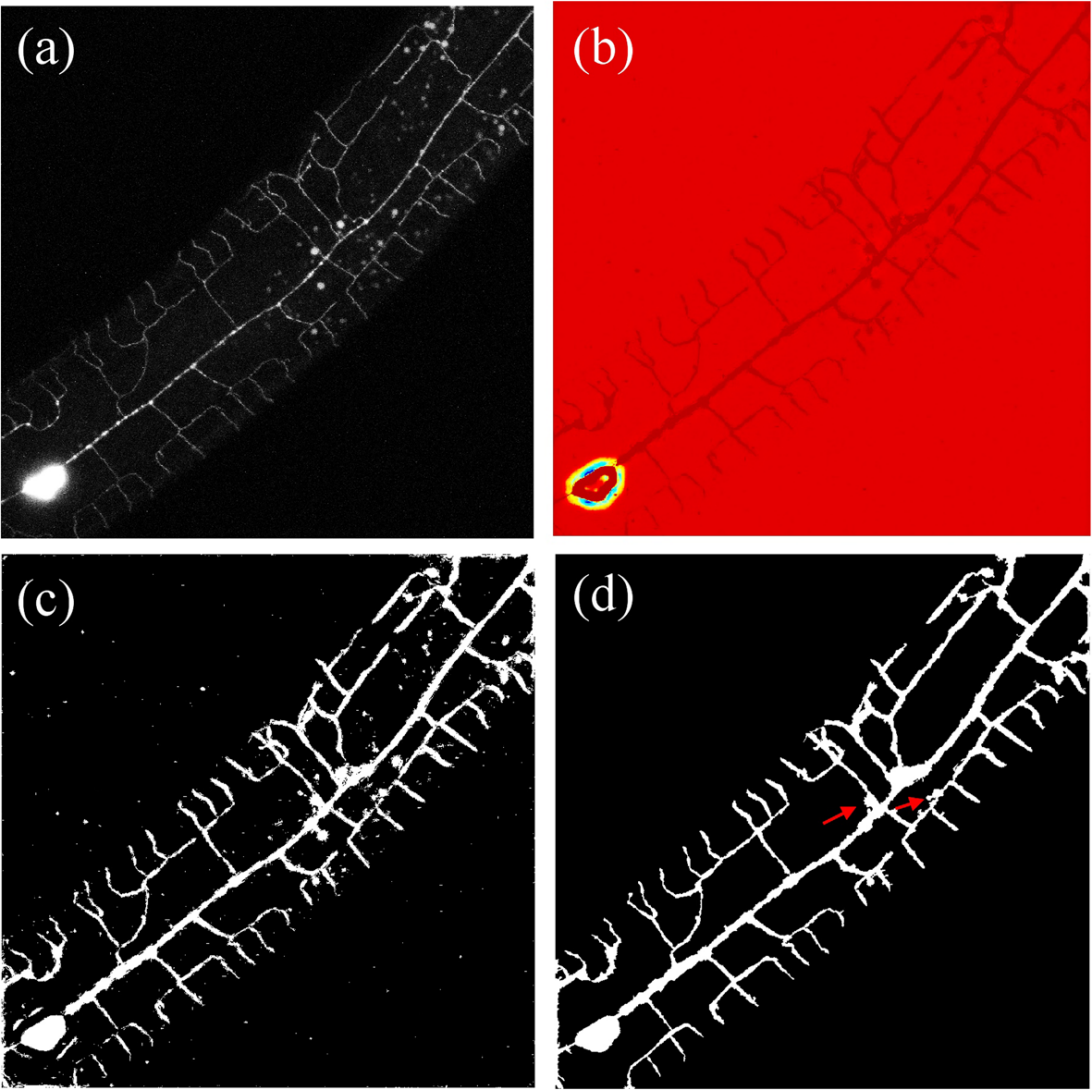
**Dendritic tree extraction (DTE) method.** For an example MIP image **(a)**, we show the outcome after computing posterior probability, Eq. (3) in **(b)**. Low values are red while higher values appear darker. **(c)** depicts the outcome after the optimized sensitivity threshold is applied to (b) to obtain a coarse segmentation. **(d)** Results after the automatic refinement and post-processing steps. Red arrows indicate examples of remaining artifacts, as a result of the presence of granules, around the cell body (soma) and tree.

As shown in Figure 3a and b, Eq. (3) assigns higher probability to regions that appear as tree-like given the models estimated from the training data. Note that we assume the prior to be equal to 1/2; this assumption follows as estimating the prior from data can be challenging since it highly depends on the experimental set-ups used and in particular on the magnification used to acquire images of dendritic trees.

While Eq. (3) provides a likelihood of a pixel being part of a tree at each image location, we are ultimately interested in a binary segmentation of the tree. To this end, we need to select an appropriate sensitivity threshold to classify each pixel as background or tree using the result of Eq. (3). To do this, we optimize a performance measure using our training set where we define our measure to be the distance from any point on the Receiver Operating Characteristic (ROC) curve [40-42] to an ideal error-less classifier, $d_{\text{corner}}=\sqrt{{\left(\text{FPR}\right)}^{2}+{\left(1-\text{TPR}\right)}^{2}}$ ($\in [0,\sqrt{2}]$), where *FPR* is the False Positive Rate (i.e., *FP* / (*FP* + *TN*)) and *TPR* is the True Positive Rate (i.e., *TP* / (*TP* + *FN*)). Note that a point on the ROC curve corresponds to a unique sensitivity threshold on *P*(*L*_*p*_ = 1|*X*_*q*_ = *x*).

Here, *TP* (True Positive), *FP* (False Positive), *FN* (False Negative) and *TN* (True Negative) refer to the elements of the Confusion Matrix [43]. Since our training sets include more than one image, counts for *TP*, *FP*, *FN* and *TN* are calculated over the entire training sets, such that they are a sum of counts from all the training images. The highest optimization score (noted F-score and defined as $\sqrt{2}-d_{\text{corner}}$, with F-score $\in \;[\;0,\sqrt{2}]$) is achieved when the distance from the upper left corner of the ROC graph is minimal. Figure 3c depicts the segmentation of a dendritic tree using the optimized sensitivity threshold.

### Post processing

Due to differences in the training and test images, the coarse extraction step typically produces spurious responses (see Figure 3c). For this reason, we proceed with a two stage post-processing step. In the first, we automatically smooth our responses by applying morphological operations of opening and closing in order to remove isolated pixels and fill missing regions. This is followed by using a Hough transform [44] in order to discriminate further lines’ structures from the coarse segmented image, while keeping only “strong" lines according to empirically-chosen thresholds. Next, we add these detected lines to the initially segmented image (i.e., after the morphological steps) to produce a cleaner segmented image featuring magnified structures of the lines; Figure 3d displays the result of such operations for an example MIP.

Finally, we perform a minor semi-automatic post-processing step where the user is given the opportunity to attempt to improve parts of the segmented dendritic tree that are not resolved to satisfaction. That is, the user can label areas that weren’t connected in the segmented image after undergoing the automatic post-processing stages and concurrently unlabel areas that were potentially misclassified as a tree. We emphasize here that the more critical step is to unlabel areas that are connected to the tree, or lie in close proximity to the tree, but do not belong to it.

## Results

We have first tested our tree extraction method across an image dataset of MIPs of mechanosensory *C. elegans* neurons (*n* = 12), obtained from fluorescence confocal microscopy. In order to image the nematodes’ dendritic trees, transgenic *C. elegans* expressing cytoplasmic ser-2p::GFP (green fluorescent protein, GFP) or plasma membrane DES-2::GFP in the PVD neurons were used. Here, *C. elegans* are anesthesized and placed between an agar pad and a *#*1.5 coverslip; for extended details on methods, see Oren-Suissa *et al.*[6].

We recall that our end goal is to provide users (e.g., biologists) with segmentations of dendritic trees from MIPs. Thus, we have manually labelled the MIP dataset to build our training set (“ground truth”) from which the model parameters for the foreground and background models are respectively estimated (i.e., $N(\cdot ;\mu_{1},\Sigma_{1})$ and $N(\cdot ;\mu_{0},\Sigma_{0})$, as introduced in Model parameter estimation), and from which the sensitivity threshold for the coarse initial segmentation is estimated from (see Algorithm Performance below).

### Cross-validation and algorithm performance

Since the training dataset is small (i.e., typical datasets for dendritic trees are on the order of <20 MIPs for a given strain, see for example [6,18,20]), we have implemented a Leave-One-Out Cross-Validation (LOOCV), where the testing subset includes only one sample while the training set includes all other samples. The process is then repeated until each image is used once as a test image; note that LOOCV is known to be nearly unbiased but costly in variance [45]. This method is however widely adopted when the number of samples is very small [46] as is the case here. In order to evaluate our algorithm’s performance, we compare our segmentation results with a traditional intensity-based thresholding method [47,48] and evaluate their overall performances against the corresponding manually-labeled segmentations.

**Figure 4:**
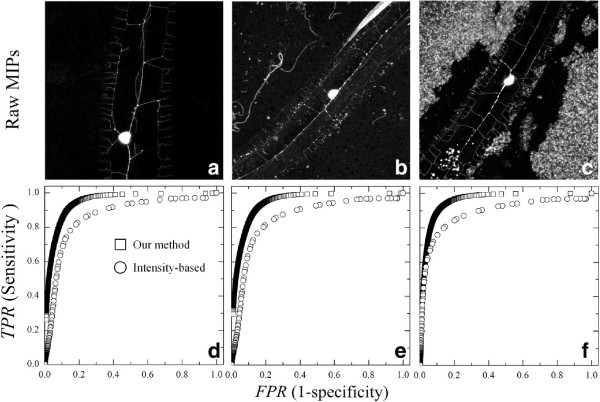
**MIPs and corresponding ROC curves of neuronal dendritic trees. (a)-(c)** Typical examples of MIP images of *C.elegans* neuronal dendritic trees. MIPs illustrate varying degrees of background noise including the presence of autofluorescent gut granules and bacteria *E. coli*, the main nutrient source for *C.elegans* (see in particular example **(c)**). **(d)-(f)** Corresponding performance ROC curves (*TPR* vs *FPR*) using both our DTE method and a traditional intensity-based thresholding method for comparison.

Figure 4 presents examples of typical MIPs of dendritic trees (a-c) and their corresponding performance ROC curves (d-f) following (i) our DTE method and (ii) the intensity-based method. Here, we qualitatively see that our method outperforms the intensity-based method. Recall that curves with both higher values of TPR and lower values of FPR are considered better, and are positioned nearer the top left corner of (i.e., TPR = 1 and FPR = 0). Following the LOOCV approach, we obtain F-scores of 1.251±0.009 (*n* = 12) for our algorithm, compared with a lower 1.160±0.012 for the intensity-based method (*p*<0.001, upon conducting a two-sample t-test). As additional validation, we compared results of our algorithm using the complete RFS filter bank [36,38], where F-scores yield 1.208±0.012 (*p* < 0.001). While implementing the complete RFS outperforms the intensity-based method, the MR8 filter bank exhibits nevertheless a tangible improvement for selecting an initial segmentation threshold. Taken independently, tree pixel intensities are generally very similar to noisy background pixels. As a result, the intensity-based method is not sufficiently accurate. In contrast, the MR8 feature set (and all RFS-derived filter banks) highlights textural cues in the image and thus emphasizes existing differences between background and foreground textures. Moreover, MR8 is rotationally invariant such that dendritic tree structures are highlighted without any bias to a specific orientation.

### Final segmentations

**Figure 5:**
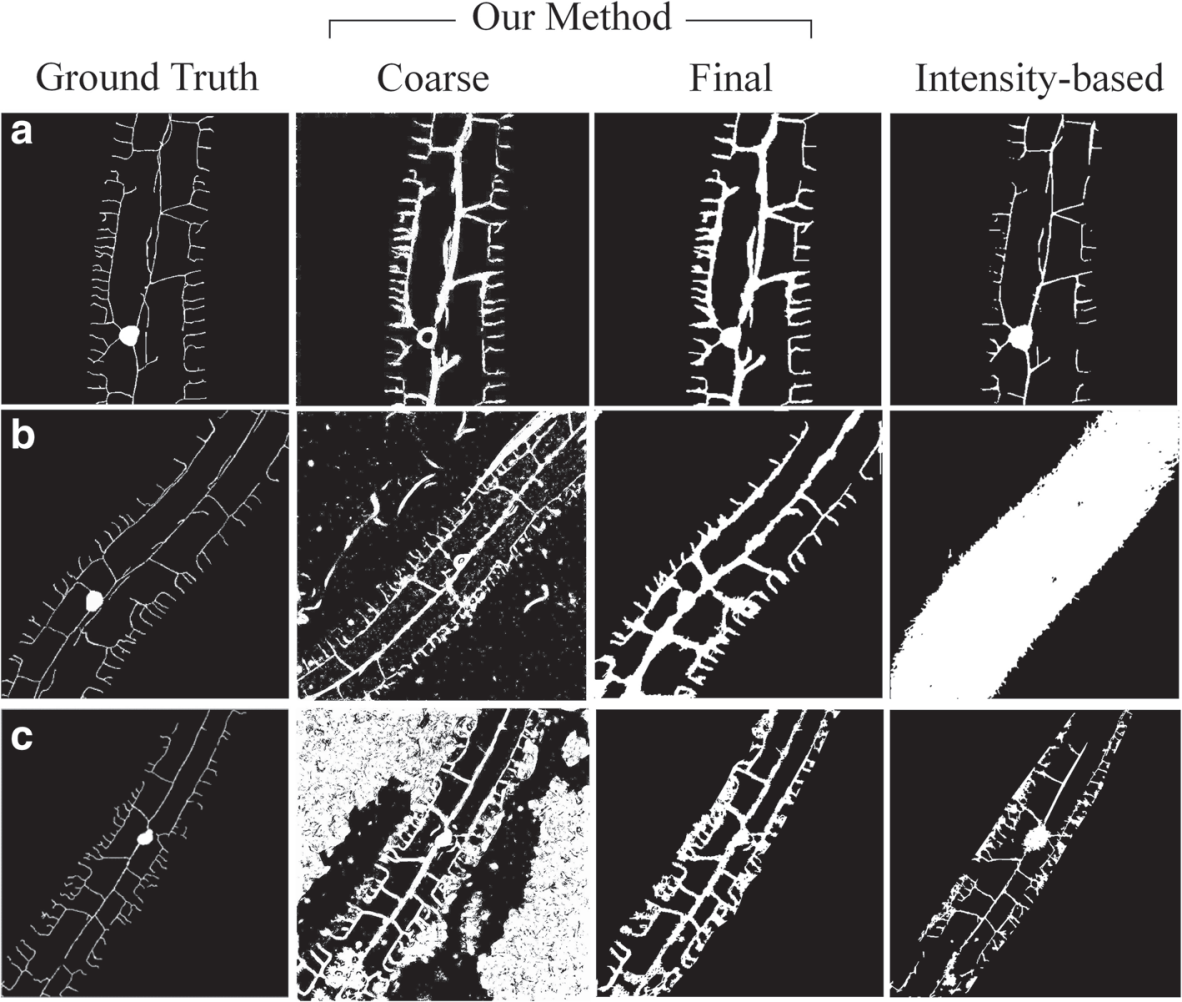
**Dendritic tree segmentations. (a)-(c)** Three example MIP images introduced in Figure 4. From left to right, columns illustrate (i) “ground truth” images from manual-labeling, (ii) coarse and (iii) final segmentations using our DTE method, compared with (iv) a traditional intensity-based method.

In Figure 5, we depict qualitative results of the final segmentations obtained for the examples shown earlier in Figure 4; initial coarse segmentations are also provided for comparison. Also shown are the corresponding “'ground truth” segmentations as well as results obtained with the traditional intensity-based method. We immediately notice that the intensity-based method achieves problematic results, in particular when considering structure connectivity, further underlining the lower F-scores obtained from the ROC curves (Figure 4). In contrast, our method provides far more suitable segmentations, visually capturing the main characteristics of the dendritic tree while largely preserving connectivity across most parts of the tree.Upon closer inspection, we notice that our segmentation method is not perfect however. For example, qualitative segmentation errors leading to artifacts are apparent in the presence of granules (see Figure 3d) or alternatively, when distant branches of the tree are covered with background noise (see Figure 5c, third column), rendering an adequate identification of these terminal branches difficult. Furthermore, we note that our DTE method delivers segmentations that intrinsically produce a tree structure with thicker branches compared to the original MIPs. Such outcomes may become problematic when branches are originally postioned closer to one another and their segmentation unites them (see Figure 5b, third column); we have attempted to address this latter issue by following a morphological “skeletonization” process (see discussion below).

**Figure 6:**
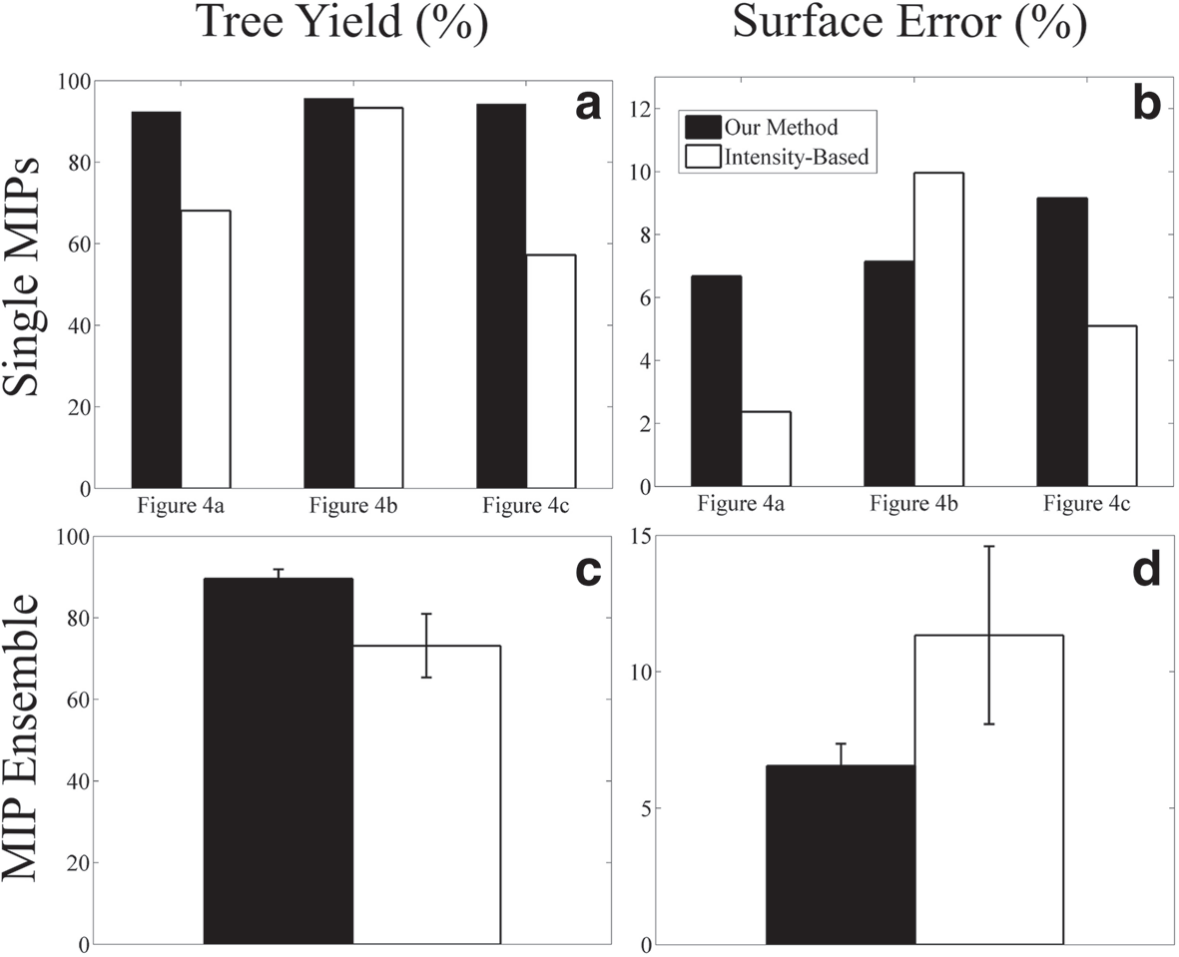
**Performance evaluation of final tree segmentations between our method and a traditional intensity-based method. (a)** Tree yield (%): proportion of the tree region that is correctly segmented for the individual MIPs of Figure 4. **(b)** Surface error (%): proportion of pixels misclassified over the entire MIP. **(c)** and **(d)** Corresponding tree yield and surface error obtained for the entire MIP dataset; mean and standard deviations are obtained across *n*=12 images.

To quantitatively evaluate and compare final segmentations using our method, Figure 6 presents the dendritic tree segmentation yield and the overall image segmentation error for the example MIPs introduced in Figure 4; these performance metrics have been recently defined [49] and are compared with the traditional intensity-based method. For each MIP, we show in Figure 6a the proportion of the nematode pixels that is correctly segmented in a given image (i.e., Tree Yield). Correspondingly, we show in Figure 6b the proportion of pixels that is incorrectly labeled over the entire image (i.e., Surface Error).

We note that the intensity-based method achieves a yield <70*%* for two of the evaluated images and a 100% yield for the image shown in Figure 6a; such high yield is however largely artificial and results from the large number of misclassified pixels belonging to the tree class, causing an ideal *TP* count. This latter observation is supported by noting the high surface error count in Figure 6b (see corresponding Figure 5b, last column), while the two other MIPs produce a significantly lower surface error (<5*%*). Our method on the other hand produces tree segmentations with a yield above 90% while maintaining a reasonable surface error (<10*%*). In Figure 6c,d, we report the corresponding tree yield and surface error across the entire MIP dataset, as produced from the best optimization score with LOOCV. Our method significantly outperforms the intensity-based method with a higher tree yield (89.55*%* ± 2.38*%* compared with 73.16*%* ± 7.80*%*) and a significantly lower error rate (6.56*%*±0.80*%* compared with 11.34*%*±3.26*%*).

### DTE and skeletonization

**Figure 7:**
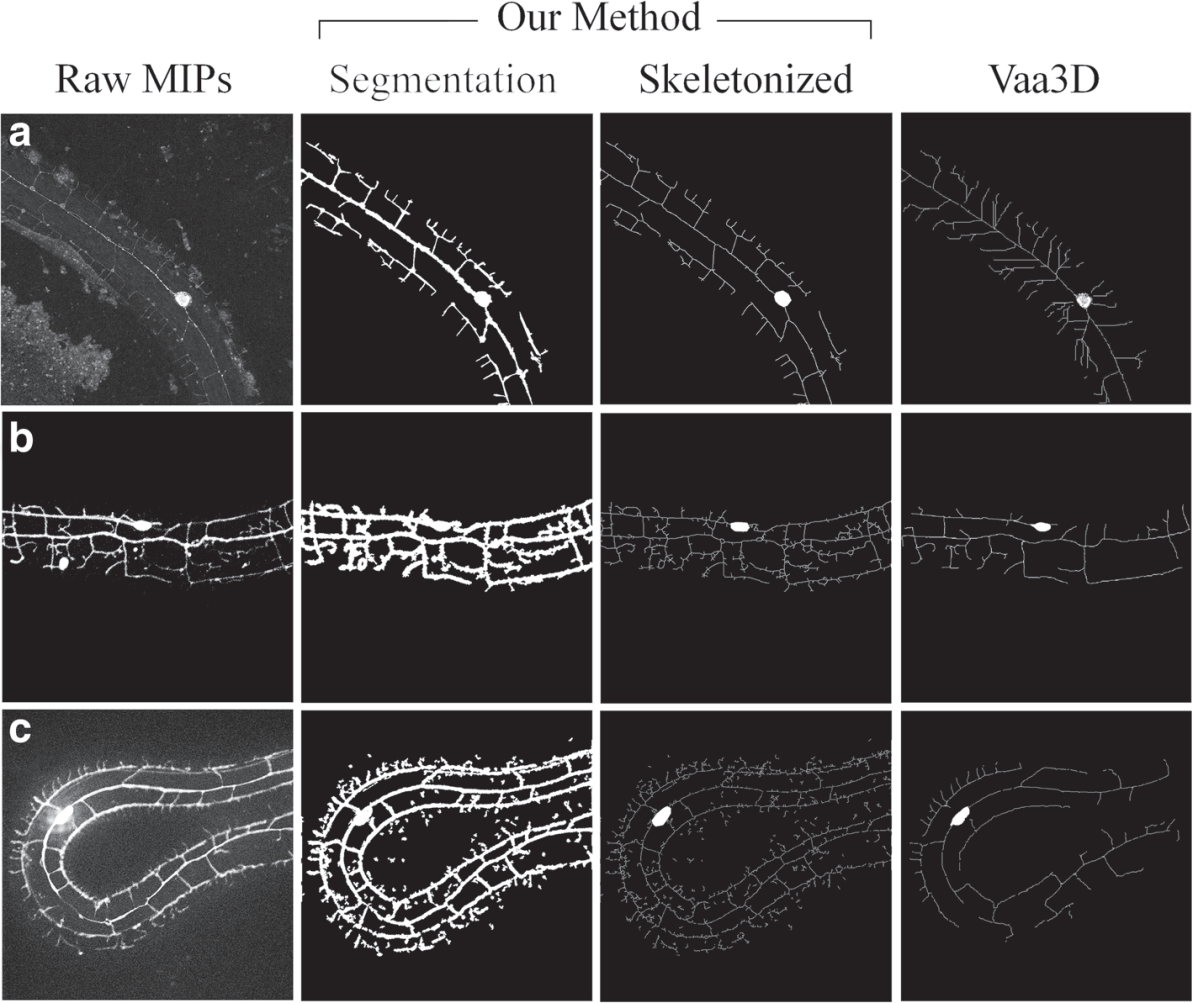
**DTE method applied to unseen MIPs.** Rows **(a)-(c)** feature three dendritic tree samples, illustrating raw images (first column), our final segmentations (second column), our skeletonized trees (third column) and traces provided by the freely available Vaa3D software [29] (last column). **(a)** through **(c)** illustrate increasing inaccuracies resulting from the skeletonization step, leading to artificial branches and loops. Note that prior to the skeletonization process, somas are manually removed to prevent additional artificial loops; the soma is automatically re-integrated after skeletonization.

To further assess the applicability of our DTE method, we apply our algorithm to examples of previously unseen MIPs (Figure 7, left column). In other words, these independent MIPs do not belong to the aforementioned MIP image dataset from which the parameters of the foreground and background noise models ($N(\cdot ;\mu_{1},\Sigma_{1})$ and $N(\cdot ;\mu_{0},\Sigma_{0})$) are respectively estimated. Note that accordingly, manually-labeled binary masks are not available for these new, independent MIPs. Using the estimated parameters of the statistical model based on our dataset (*n* = 12), we qualitatively observe that in general our DTE method yields satisfactory results for final segmentations (Figure 7, middle left columns).

In view of further data extraction for general tree morphology characterization [6], we have attempted to skeletonize segmented dendritic trees following a morphological “Thinning” process [50]. Obtaining such skeletons often allows easier viewing of the dendritic tree structure that can be beneficial for potential users. Sample results for the independent MIPs are illustrated in Figure 7 (middle right column). Here, the thinning operation is calculated by translating the origin of a structuring element to all possible pixel positions in the image. Then, at each pixel position the underlying image pixels are compared to the structuring element’s pixels. If the foreground and background pixels (i.e., pixels with a binary value of 1 or 0, respectively) in the structuring element match identically foreground and background pixels in the image, the image pixel underneath the origin of the structuring element is set to be background (i.e., to zero); otherwise it is left unchanged [48]. While the skeletonization process delivers a compact representation of the tree (Figure 7a), it may also yield as a result of irregularities in the final segmented structures, spurious features such as artificial branches, loops and gaps in the last dendritic generations (see Figure 7b,c), while the main branching structures remain clearly identifiable. Nevertheless, the skeletonized representation of dendritic trees is generally attractive for further morphological quantification.

For comparison, we also illustrate in Figure 7 (last column) how “Vaa3D-Neuron1”, a freely-available [51] state-of-the-art tracing software [29], traces the corresponding dendritic tree images when a user provides manually a number of required seed points along each tree leaves and bifurcations; results shown in Figure 7 (last column) correspond to approximately 120 annotations per image. Briefly, this tracing algorithm attempts to deliver tree tracings that pass through each seed annotation provided by the user. We note that while Vaa3D is capable of tracing out the general structure of the dendritic trees, it provides however numerous spurious branches and connections (e.g., first row of Figure 7). Overall, we observe that our DTE approach (following the skeletonization process) provides qualitatively the same if not better dendritic tree tracings with significantly less manual involvement on the user end.

## Conclusion

We have presented a statistical computer vision method for segmenting dendritic tree structures from MIP images of *C. elegans*. Our approach makes use of texture-based features that are invariant to orientation changes in an effort to characterise noisy tubular-like image patches. These features are then used in a probabilistic model that provides a coarse tree segmentation before further fine-tuning using post-processing steps. We quantitatively show that our method delivers reliable segmentations for various noisy MIP imaging scenarios and widely outperforms traditional intensity-based methods. In addition, we show qualitatively that our method performs at least as well, if not better to more sophisticated methods when extracting the dendritic tree outline. Altogether, our DTE method is anticipated to help unburden manual labor on the user end and to appeal to a growing community of researchers interested in characterization of neuronal arborization in *C. elegans*.

## Competing interests

The authors declare that they have no competing interests.

## Author’s contributions

MO and BP initiated the project. AG, RS and JS designed the statistical methods and segmentation algorithms. PA and BP provided critical insight and JS supervised the project. AG, RS, PF and JS drafted the manuscript. MS conducted the confocal imaging. All authors read and approved the final manuscript.
